# Morphological modularity in the vertebral column of Felidae (Mammalia, Carnivora)

**DOI:** 10.1186/s12862-017-0975-2

**Published:** 2017-06-09

**Authors:** Marcela Randau, Anjali Goswami

**Affiliations:** 1 Department of Genetics, Evolution and Environment, University College London, Darwin Building, Gower Street, London, WC1E 6BT UK; 2 Department of Earth Sciences, University College London, Gower Street, London, UK

**Keywords:** Integration, Two-block PLS, Axial skeleton, Vertebra, Carnivora, Modules, Geometric morphometrics

## Abstract

**Background:**

Previous studies have demonstrated that the clear morphological differences among vertebrae across the presacral column are accompanied by heterogeneous functional signals in vertebral shape. Further, several lines of evidence suggest that the mammalian axial skeleton is a highly modular structure. These include its composition of serial units, a trade-off between high shape variance and strong conservation of vertebral count, and direct association of regions with anterior expression sites of *Hox* genes. Here we investigate the modular organisation of the presacral vertebral column of modern cats (Felidae, Carnivora, Mammalia) with pairwise comparisons of vertebral shape covariation (i.e. integration) and evaluate our results against hypotheses of developmental and functional modularity. We used three-dimensional geometric morphometrics to quantify vertebral shape and then assessed integration between pairs of vertebrae with phylogenetic two-block partial least square analysis (PLS).

**Results:**

Six modules were identified in the pairwise analyses (vertebrae included are designated as ‘C’ for cervical, ‘T’ for thoracic, and ‘L’ for lumbar): an anterior module (C1 to T1); a transitional module situated between the last cervicals and first thoracics (C6 to T2); an anterior to middle thoracic set (T4 to T8); an anticlinal module (T10 and T11); a posterior set composed of the last two thoracics and lumbars (T12 to L7); and a module showing covariation between the cervicals and the posterior set (T12 to L7). These modules reflect shared developmental pathways, ossification timing, and observed ecological shape diversification in living species of felids.

**Conclusions:**

We show here that patterns of shape integration reflect modular organisation of the vertebral column of felids. Whereas this pattern corresponds with hypotheses of developmental and functional regionalisation in the axial skeleton, it does not simply reflect major vertebral regions. This modularity may also have permitted vertebral partitions, specifically in the posterior vertebral column, to be more responsive to selection and achieve higher morphological disparity than other vertebral regions.

**Electronic supplementary material:**

The online version of this article (doi:10.1186/s12862-017-0975-2) contains supplementary material, which is available to authorized users.

## Background

Numerous studies have demonstrated that organisms can be partitioned into sets of phenotypic traits or structures that show coordinated patterns of variation or evolution. These sets of traits, termed phenotypic modules, can be defined as units composed of multiple traits that display high levels of covariation with other traits within that unit, but relatively weak covariation with traits outside of the unit. The related concept of integration refers to the overall magnitude of covariation of phenotypic traits, and can refer to a single module, which would be expected to display relatively high within-module integration, or may span multiple modules or structures [1–3]. The integration of traits, and their organisation into discrete phenotypic modules, has been hypothesised to arise and/or evolve as a product of shared developmental origin or pathways, genetic pleiotropy, or common function [1, 2, 4, 5]. Strong integration within modules, and reduced integration between modules, is further hypothesised to promote coordination among functionally-related traits, while allowing independence and differential specialization of distinct modules [2, 6–9]. With its serial organisation and composition of vertebral units, distinguishable morphological differences among regions (cervical, thoracic, and lumbar), and direct association of those regions with expression sites of genes in the *Hox* family, the presacral axial skeleton would appear to encapsulate the concepts of regionalisation and modularity [4, 10–14].

Although regionalisation of the vertebral column can be observed in amniotes in general [10], the mammalian axial skeleton shows the greatest differentiation in regional vertebral shape [10, 15–20]. This increased divergence is accompanied by strict constraints in regional vertebral number, particularly in the cervical region with seven vertebrae present in almost all of the ~5000 mammalian species. Total presacral vertebral count is also highly conserved [21–23], although some variation does occur [24]. This invariability with regards to vertebral count has been suggested to signal strong canalisation (i.e. limitation of variation between individuals due to the tendency of organisms to “follow predetermined developmental pathways in spite of environmental and genetic perturbations” [25], page 44, and also see [26]) and developmental stability in the axial skeleton, and is thought to have evolved early in mammalian history [22, 23]. Additionally, rather than being the target of selection themselves, highly fixed vertebral numbers in mammals may reflect developmental constraints related to the muscularisation of the diaphragm and the advantages of involving the lumbar region in abdomen expansion during inspiration and in sagittal bending during locomotion [6, 22].

In addition to the almost universally fixed count of seven vertebrae in the cervical region in mammals, species of the order Carnivora also show little variation in thoracolumbar count, generally between 19 and 20 vertebrae [21]. Moreover, some families, such as Felidae (i.e. cats), display absolutely no variation in vertebral numbers between taxa: all felid species present 27 presacral vertebrae which are traditionally divided into the three main vertebral column regions (i.e. cervical, thoracic, and lumbar) by clear morphological differences [15, 27–30]. In accordance with the observed trade-off between vertebral count invariability and high morphological disparity, both linear and landmark-based analyses of vertebral shape have shown evident functional regionalisation in the axial skeleton of felids. These analyses revealed regions which differ in magnitude of phylogenetic and ecological signal (e.g. specialisation related to locomotor mode) and both ontogenetic and evolutionary allometric scaling [29, 31, 32]. Specifically, the highest covariation between vertebral shape and prey size choice or locomotory mode (i.e. the two main ecological categories that have been used to describe felid ecology in the literature [33–39]) were found in the posterior region of the vertebral column, composed of the vertebrae caudal to the posterior attachment of the diaphragm, from T10 to L7. Conversely, vertebrae in the cervical region displayed high phylogenetic signal and little significant ecological signal [29, 31].

These examples of conspicuous morphological and functional regionalisation are strong indicators of modularity in the vertebral column, and not surprisingly, modularity has indeed already been described, or at least suggested, at different levels within the mammalian axial skeleton (e.g. [4, 29, 40]). One example of a hypothesised vertebral module is composed of the mid-cervicals C3 to C5. These vertebrae, whose somites have migratory muscle precursor cells which are committed to diaphragm transformation, have been suggested to be involved in the muscularisation of the septum and consequent fixed cervical number across almost all mammals [22].

A larger hypothesised module stems from the relatively fixed count of total thoracolumbar vertebrae and has been suggested to arise from close association of these two regions, with any changes in regional vertebral number being counteracted by the inverse change in the opposite series, and thus no change to the total count (i.e. homeotic changes) [4, 11, 21, 23, 40, 41].

Our previous studies of vertebral shape evolution in felids have already suggested some hypotheses of modularity specific to this study system. The observation of regionalised patterns of allometric scaling in a linear morphometric study both supported the mid-cervical vertebral module and suggested the presence of three additional modules: an anterior cervicothoracic module, a lumbar module, and a functional ‘anticlinality module’ composed of the T10-T12 vertebrae [29]. Additionally, we have previously demonstrated that presacral vertebral shape in felids is driven by the developmental origins of vertebral components, with two morphological modules found in adult vertebral shape: the ‘centrum’ and the ‘neural spine-related’ modules (referred to as the ‘developmental two-module model’ therein to reflect the different somitic origins of these modules; [27, 40, 42]). Interestingly, this model of modularity, although widespread through most the presacral column, was not supported in vertebrae that are positioned immediately at or adjacent to the borders of morphological vertebral column regions: specifically, C4, T1, T8, L6 and L7. This observation led to the suggestion of a disruption of developmental modularity – or a functional overprint – in order to maintain the larger modular organisation of the vertebral column [40].

**Fig. 1:**
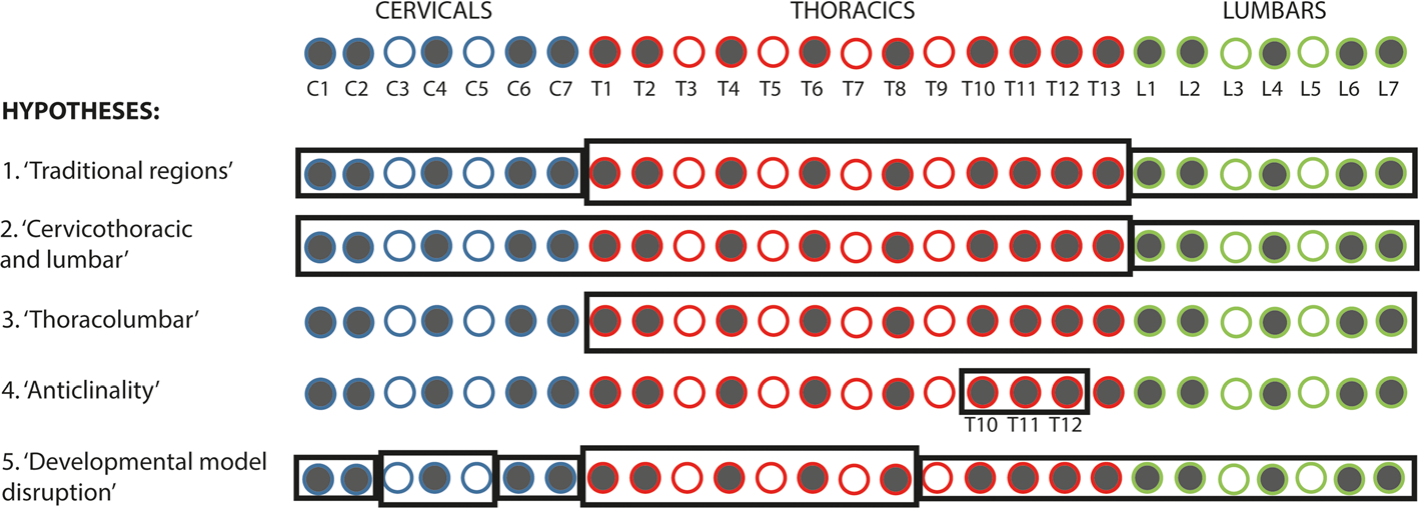
Illustration of the five hypotheses of intervertebral phenotypic modularity tested here. *Black rectangles* illustrate sets of vertebrae which are hypothesized to show high integration among themselves and, therefore, to represent a module. See text for detailed description of hypotheses. Hypothesis 1: Traditional regions. Hypothesis 2: Cervicothoracic and lumbar modules. Hypothesis 3: Thoracolumbar module. Hypothesis 4: Anticlinality model composed of vertebrae T10, T11 and T12. Hypothesis 5: Developmental model disruption. C, T, and L stand for cervicals (*blue outline*), thoracics (*red outline*), and lumbars (*green outline*), respectively. *Filled circles* describe landmarked vertebrae

Although there have been recent additions to the literature on the morphological, biomechanical and developmental changes to the vertebral column in mammals or across vertebrates in general [6, 10, 40, 43–47], much is yet unknown on its evolution and how patterns of trait integration or modularity may affect its response to selection [48]. Here we analyse patterns of shape covariation across the presacral vertebral column in order to quantify the modular organisation of the axial skeleton in felids. Specifically, we use three-dimensional geometric morphometrics to describe presacral vertebral shape and quantify intervertebral integration with pairwise comparisons of presacral vertebrae using phylogenetic two-block partial least square analysis (PLS). The results of the pairwise PLS analyses were used to test whether specific sets of vertebrae show higher magnitude of shape integration (i.e. greater covariation) within the set than with vertebral units outside of the set, therefore forming a ‘module’ [1–3]. The hypothesised intervertebral modules assessed with pairwise PLS results were drawn from the literature and are as follows (Fig. 1): 1) the ‘traditional regions’ hypothesis: Traditional regional boundaries (i.e. cervical, thoracic and lumbar) in the felid vertebral column form discrete morphological modules [4, 6, 21, 23, 30]; 2) the ‘cervicothoracic and lumbar modules’ hypothesis: Two modules composed of multiple vertebrae that share a common allometric pattern [29] can be found in the presacral axial skeleton: an anterior cervicothoracic module (where vertebrae show positive allometry related to centrum and neural spine dimensions) and a lumbar module (with positive allometry of traits related to the neural spine lever arm) [29]; 3) the ‘thoracolumbar’ hypothesis: Thoracic and lumbar vertebrae show high covariation [4, 11, 21, 23, 41]; 4) the ‘anticlinality’ hypothesis: Vertebrae T10 to T12 compose an ‘anticlinality module’ [29]; and 5) the ‘developmental model disruption’ hypothesis: Boundaries of modular organisation of the vertebral column match vertebral positions where the intravertebral developmental two-module (centrum and neural spine) model is not supported, specifically at the edges of the C3 – C5 cervical module, between cervicals and thoracics (i.e. at T1), the division of the vertebral column into pre- and postdiaphragmatic regions at T8, and at the last two presacral vertebrae L6 and L7 [40].

We further conducted separate analyses of intervertebral integration for the two intravertebral developmental modules (centrum and neural spine). Specifically, the same pairwise phylogenetic PLS analyses were conducted across the presacral vertebral column, but traits were limited to those from either the neural spine or the centrum [27, 40, 42]. Following from our previous results showing the widespread developmental two-module model of intravertebral covariation, this latter analysis allows us to assess if the patter of intervertebral covariation across the vertebral column is the same for the whole vertebral morphology and for when only trait units regarding each of these modules are considered (Additional file 1: Tables S1 and S2 for landmarks’ identity, following [40]).

## Results

### Vertebral shape covariation

Phylogenetic PLS analysis demonstrated that 108 out of the total 171 pairwise analyses were not significant (*p*-value >0.05, Table 1 and Additional file 1: Table S4), suggesting extensive modularity of the presacral vertebral column. The remaining 63 significant pairwise analyses allowed for identification of sets of vertebrae which presented particularly strong within-group covariation (i.e. PLS covariation >0.90, *p*-value <0.05). According to these results, six sets of highly covarying vertebrae were identified as follows: 1) C1 to T1; 2) C6 to T2; 3) T4 to T8; 4) T10 to T11; 5) T12 to L7; and 6) a set showing covariation between C1 to C7 (with the exception of C4) and T12 to L7, with the exception of the pairwise comparisons between C1 and the lumbars L4 and L6, and C6 and L7.

**Table 1:**
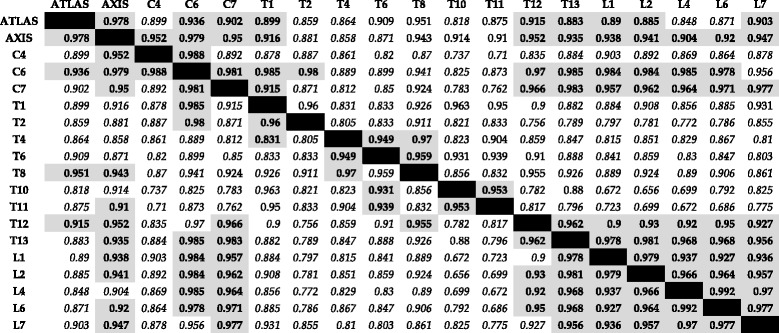
Results of phylogenetic Partial Least Squares analysis of all landmarks. Above diagonal cells show the pairwise covariation values (i.e. degree of integration, displayed as the correlation between blocks) between each pair of vertebrae, while below diagonal values display the covariation values with significance levels after Benjamini-Hochberg correction. Results in bold and in grey shaded cells show significant covariations and suggested modules, while results in italics and with white shaded cells are not significant (*p*-value >0.05, Additional file 1: Table S4)

After Benjamini-Hochberg correction (multiple comparisons correction, see Material and Methods; Table 1 and Additional file 1: Table S4), the number of covariation tests that were not significant increased to 113, leaving 58 significant results. Those tests that were rendered not significant after this correction were concentrated between the first cervicals (C1 – C4) and C7 and T1, C1 and the end-thoracics and lumbars, and some of the covariation results between the pre-diaphragmatic thoracics (i.e. thoracic vertebrae between T1 and T8). Thus, the overall pattern of intervertebral modularity was similar after correction for multiple comparisons.

### Covariation across centrum versus neural spine modules throughout the vertebral column

*Centrum:* Results from the phylogenetic PLS on centrum-only landmarks supported modules largely similar to those found when whole vertebral morphology was analysed: 1) C1 – T2, with three exceptions in pairwise comparisons between C4 and T1, C6 and C7, and C6 and T1, formed a cervical and first thoracics set; and 2) T12 to L7 composed a set with very strong within module covariation (i.e. > 0.95; Table 2 and Additional file 1: Table S5). However, other vertebral combinations were also apparent: 3) T6 - L6 vertebrae; 4) Between C1 – C4 and T8 – L4, with the exception of T11, which only presented significant shape covariation with C1 and C7 among the cervicals; and 5) C7 and every other vertebra included in this analyses, with the exception of C6.

**Table 2:**
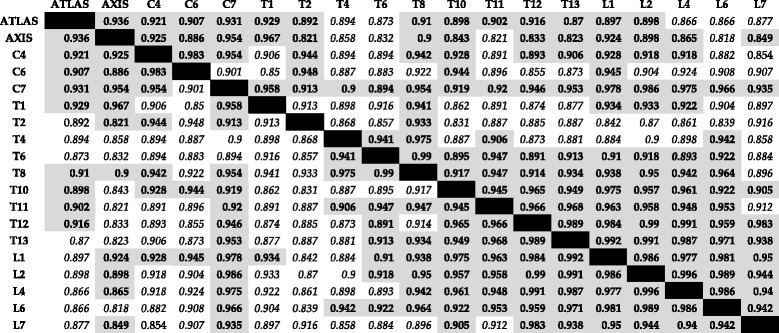
Results of phylogenetic Partial Least Squares analysis of landmarks concerning the ‘centrum’ module. Above diagonal cells show the pairwise covariation values (i.e. degree of integration, displayed as the correlation between blocks) between each pair of vertebrae, while below diagonal values display the covariation values with significance levels after Benjamini-Hochberg correction. Results in bold and in grey shaded cells show significant correlations and suggested modules, while results in italics and with white shaded cells are not significant (*p*-value >0.05, Additional file 1: Table S5)

Correction of this analysis’ significance level with the Benjamini-Hochberg procedure reduced and rendered non-significant most pairwise comparisons between C1 – C6 and T12 – L4, but had little effect on most other modules (Table 2 and Additional file 1: Table S5).

### Neural spine

There were fewer significant pairwise covariation results from the phylogenetic PLS on neural spine-only landmarks than from the centrum-only analysis (i.e. 76 versus 114 significant covariation results prior to correction for multiple comparisons, respectively; Table 3 and Additional file 1: Table S6). The significant pairwise tests on neural spine-only landmarks displayed four distinct modules: 1) between C1 and C7, with the exception of C4; 2) between T10 and T11; 3) between vertebrae in the T12 – L7 region; and 4) between the cervicals C1 – C7, with the exception of C4, and T12 – L7. Benjamini-Hochberg correction did not change these patterns and mainly reduced the covariations between the cervicals and the vertebrae in the T12 – L7 region and other vertebral pairs in the thoracic region (Table 3 and Additional file 1: Table S6).

**Table 3:**
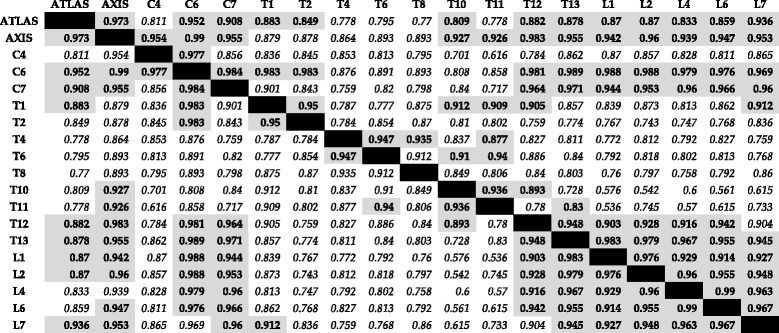
Results of phylogenetic Partial Least Squares analysis of landmarks concerning the ‘neural spine’ module. Above diagonal cells show the pairwise covariation values (i.e. degree of integration, displayed as the correlation between blocks) between each pair of vertebrae, while below diagonal values display the covariation values with significance levels after Benjamini-Hochberg correction. Results in bold and in grey shaded cells show significant covariations and suggested modules, while results in italics and with white shaded cells are not significant (*p*-value >0.05, Additional file 1: Table S6)

## Discussion

The results presented here provide new information on the structural organisation of the vertebral column in felids, and potentially mammals in general. In light of the results presented here, the ‘traditional regions’ hypothesis (i.e. ‘the cervical, thoracic and lumbar regions in the felid vertebral column form discrete morphological modules’) and the ‘cervicothoracic and lumbar modules’ hypothesis (i.e. ‘two modules composed of multiple vertebrae that share common allometric patterns: an anterior cervicothoracic and a lumbar module’) could be rejected or considered insufficiently explanatory. Although high covariation was found between vertebrae within each of these regions, those either did not include all or most vertebrae which compose the regions or, more commonly, sets of highly covarying vertebral shapes were inclusive of vertebrae beyond the traditional boundaries. Specifically, in all of the analyses performed, with the exception of the phylogenetic PLS of the neural-spine landmarks, covariation in the anterior portion of the axial skeleton included high pairwise covariation between cervicals and the first thoracics. Additionally, all cervicals analysed here, with the exception of C4 displayed high covariation with the last thoracics and lumbar vertebrae.

A distinct module composed of vertebrae in the cervicothoracic boundary (i.e. C6 – T2) was found. A developmental covariation had already been suggested for these units based on the migration of cells from somites bound to the forelimbs, which may additionally have been involved in the first evolutionary steps that contributed to the muscularisation of the diaphragm [22]. Due to the lack of vertebrae C3 and C5 in our dataset, it was not possible to test for higher covariation between those and C4, composing the suggested C3 – C5 developmental module in mammals [22]. Nevertheless, C4 presented very high covariation with both C2 and C6, indicating that a C3 – C5 set would likely not be distinguishable as a separate morphological module in the analyses presented here.

High covariation was found between the two last thoracics, T12 and T13, and the lumbars. These two last thoracic vertebrae indeed have morphological characteristics that resemble lumbar shape more than they do the rest of the thoracics, such as a larger centrum, a cranially oriented neural spine and the presence of accessory processes [30, 31, 49]. This result thus supports the ‘thoracolumbar’ modularity hypothesis (i.e. ‘thoracic and lumbar vertebrae show high covariation’), although only with regards to these last thoracics. Additionally, when considering mammals in general, this T12 – L7 modularity could facilitate, or be driven by, the homeotic changes between the thoracic and lumbar regions which can promote vertebral column variation without changes in overall vertebral count [4, 6, 21, 23, 30].

We found strong support for the ‘anticlinality’ hypothesis [29], although this was only composed of vertebrae T10 and T11, and not T12. This group comprises a biomechanically important region of the axial skeleton for two main reasons. Firstly, T10 is the diaphragmatic vertebra, which marks the dorsocaudal attachment of this septum and is also the first of the thoracic vertebrae to present ribs which are vertebrochondral, commonly named ‘false’ or ‘floating’, instead of vertebrosternal ribs (i.e. vertebrochondral ribs attach to cartilages of another rib instead of directly to the sternum) [30]. This release from the physical constraint of direct attachment allows for greater sagittal bending towards the posterior end of the vertebral column, particularly in the rib-less lumbar region [6, 11, 21, 32]. Secondly, T11 is the anticlinal vertebra, with a much reduced and usually perpendicular neural spine, marking the change in neural spine orientation from a caudally inclined process prior to this vertebra to the cranially orientated process present in vertebrae T12 through L7 [29–31, 49]. This change in neural spine orientation is especially well defined in carnivorans (specifically in Canidae and Felidae) and, along with the observed increase in centrum length, promotes greater motion and sagittal bending of the posterior region of the axial skeleton [29, 31, 49].

**Fig. 2:**
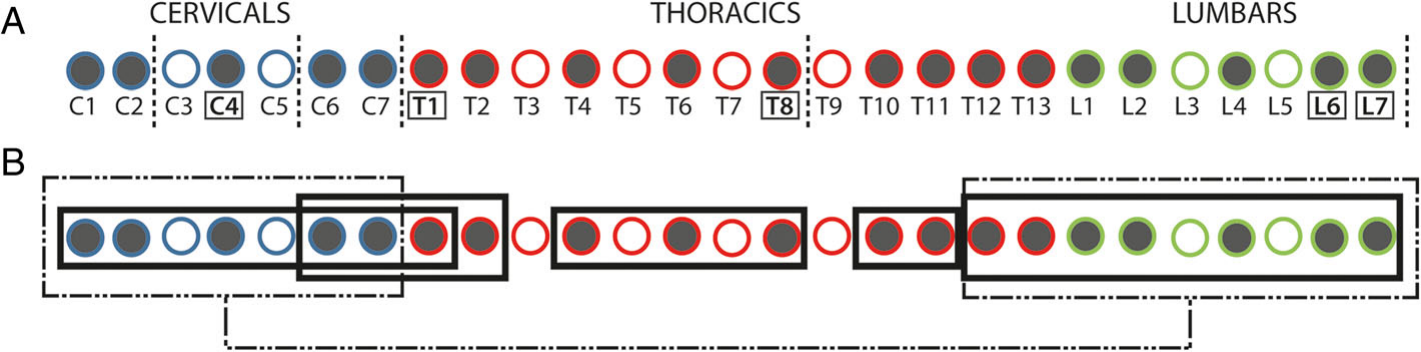
Schematics of vertebral column modules based on pairwise covariation between vertebrae. **a** *Vertical dashed lines* are hypothesized regional boundaries based on vertebrae showing disruption of the two-module model for intravertebral shape covariation (vertebrae C4, T1, T8, L6, and L7; [40]). **b** *Rectangular boxes* showing suggested vertebral column modules. *Dashed boxes and connecting line* describe covariation between the cervicals and the T12 – L7 vertebrae. C, T, and L stand for cervicals (*blue outline*), thoracics (*red outline*), and lumbars (*green outline*), respectively. *Filled circles* describe landmarked vertebrae

Finally, the boundaries of the modules found here mostly supported the ‘developmental model disruption’ hypothesis, in which it was proposed that boundaries of intervertebral modules would reflect the positions of vertebrae that did not show significant intravertebral modularity [40]. The intravertebral developmental modularity model of two modules was not supported in vertebrae C4, T1, T8, L6 and L7 [40], and the results presented here show that most of the intervertebral modules follow the hypothesized boundaries or have vertebral boundaries that only slightly differ from those by one vertebra (Fig. 2). This result is best displayed in the mid-posterior region. Anterior to the suggested boundary at T10 between the prediaphragmatic and postdiaphragmatic vertebrae, the T1-T8, or mid-thoracics T4 – T8 composed a distinct set; while the postdiaphragmatic vertebrae were divided into two modules (T10 – T11, and T12 – L7) with very high within-module covariation. As discussed above, these postdiaphragmatic vertebrae undergo more pronounced bending due to the release from the physical constraints of the ribs and diaphragm [29, 31, 49]. Accordingly, previous studies have shown that the T10-L7 region shows higher ecological signal in felids and that measurements from this region are best at separating species in a vertebral morphospace [29, 31]. Furthermore, our previous study has shown that these vertebrae also displayed the greatest overall intravertebral integration and morphological variance, an observation which supports the hypothesis of high integration being able to facilitate increased levels of disparity, and therefore promoting morphological evolution on those preferred axes of variation (i.e. “lines of least resistance” hypothesis) [40, 48, 50]. Taken together, these results indicate that the postdiaphragmatic vertebrae T10 –L7 compose an evolutionarily highly responsive region which is organised into two strongly covarying modules. This modularity may therefore be responsible for maintaining the organisation and relative independence of this region, while the high integration both within each module and within individual vertebrae may contribute to higher levels of shape disparification (and ecological specialisation) while retaining functionality.

In the anterior vertebral column, support for the ‘developmental model disruption’ hypothesis is less clear, as the first two cervicals were not supported as a separate module. However, C4, which did not support the two-module developmental model in our analysis of intravertebral modularity [40], only displayed significant covariation with two of the other analysed vertebrae. Additionally, two well-supported modules were found either near or involving the suggested boundary between the last cervical and first thoracic: a module composed of C1 – T1, and another of C6 – T2. As discussed above, these vertebrae have been suggested to be highly constrained by development [6, 21, 22], and show significant phylogenetic signal, but no ecological signal, in shape across felids [31].

The analyses presented here revealed surprisingly strong covariation between the most anterior and most posterior presacral vertebrae (C1 – C7 and T12 – L7 in the phylogenetic analysis, Table 1). This result was unexpected as we had hypothesized higher covariation between more thoracic and lumbar vertebrae instead [4, 51]. However, the origin of this pattern may lie in vertebral ossification timing. A study of ossification sequences in the domestic cat skeleton [27] reported that thoracic elements developed prior to both the cervical and lumbar regions. In this case, this shared later ossification of cervical and lumbar vertebral elements could relate to the observed covariation of these two regions. Additionally, a more recent study of vertebral ossification in 17 species of mammals [43] (including one monotreme, six marsupials and ten placentals, but not including any felids) has shown that, although neural arches ossify first and begin ossification in the first cervicals and first thoracics, these are followed by ossification in the other cervicals and lumbar regions. Subsequently, centra ossify first in the thoracic region and ossification spreads both cranially and caudally [43]. This progression of centra ossification in both directions could indeed cause a coincidence in ossification timing in cervicals and posterior T2 – L7. While this potential explanation for the pattern of covariation among these two regions is speculative, it could be tested with more detailed ossification sequence data from felids, vertebral modularity studies across mammals, and biomechanical analyses of the axial skeleton across felids and other mammals.

The results from the phylogenetic PLS on centrum or neural-spine-related coordinates also offer some support to this new hypothesis of integration between cervicals and T12-L7, tentatively due to ossification timing (Tables 2 and 3). There was a clear and strong association between the neural-spine landmarks of cervical vertebrae (with the exception of C4) and vertebrae in the T12 – L7 region. This covariation was slightly less consistent but still present in the analysis of the centrum-related landmarks, although in this case the atlas (C1) and C6 also displayed fewer covariations with posterior vertebrae in addition to C4. Additionally, those posterior vertebrae with significant covariation were generally the more anterior ones, from T8 – L4, with the exception of T11, reflecting the direction of centrum ossification. However, we would expect a stronger signal of this covariation in the centrum landmarks, rather than the neural spine landmarks, contrary to our results.

The separate centrum and neural-spine analyses also supported the other modules found in the PLS analyses of whole vertebrae. Results from centrum-only landmarks showed modularity of the vertebral column into an anterior cervicothoracic module from C1 – T2, with five pairwise exceptions between axis (C1) and T2, C4 and T1, C6 and C7, C6 and T1, and C6 and T2. This analysis also showed stronger interaction between the thoracics and lumbars, with a strong T6 – L6 module, and among vertebrae in the T12 – L7 module. Neural-spine traits further supported this T12 – L7 module, as well as the C1 – C7 module (with C1 and C4, C2 and C4, and C4 and C7 as exceptions), and the anticlinality T10 – T11 module.

## Conclusions

Here we have performed an empirical analysis of intervertebral integration and compared our results to previously suggested hypotheses of developmental and functional modularity across the presacral vertebral column. Our results demonstrate that modularity is prevalent in the axial skeleton of felids, but that modules do not necessarily agree with the traditional regions of cervical, thoracic and lumbar vertebrae. Instead, vertebral morphological modules reflect five main groupings which organise the vertebral column according to either developmental constraints or function. Those regions have also been shown to differ considerably in their morphological disparity, phylogenetic signal, and ecological specialisation, and have been suggested to present opposing levels of evolvability. Additionally, the observed interaction between the cervicals and lumbars may reflect their shared ossification timing. Finally, the recovered modules supported the hypothesis that overall modularity of the vertebral column reflects the positions of the few vertebrae which show disruption of the intravertebral developmental two-module model. Specifically, the few vertebrae in which the developmental two-module model was not supported form the boundaries of the intervertebral modules found here.

Although this study is limited to a subset of representatives from a single family, the similarities in the modular organisation found here to developmental patterns shared across mammals suggest that these results may reflect a common mammalian condition. Importantly, the modular organisation of the vertebral column demonstrated here highlights that both development and function are important factors shaping vertebral shape diversification. Therefore, it may be the trade-off between these influences that control the disparity observed in the axial skeleton across mammalian families.

## Methods

An Immersion Microscribe G2X (Solution Technologies, Inc., Oella, Maryland) was used to collect three-dimensional (3D) landmarks on 19 out of the 27 felid presacral vertebrae. These 19 vertebrae comprised the atlas (C1), axis (C2), C4, C6, C7, T1, T2, T4, T6, T8, T10, T11, T12, T13, L1, L2, L4, L6, and L7 (where C stands for cervical, T for thoracic, and L for lumbar). Reasons for vertebrae selection have been detailed extensively in previous studies [29, 31, 40]; in short, the chosen vertebrae cover the observed range in presacral vertebral morphology and include vertebrae which compose the boundaries between traditional vertebral morphological regions (e.g. C7 and T1 forming the boundary between the cervical and thoracic regions).

**Fig. 3:**
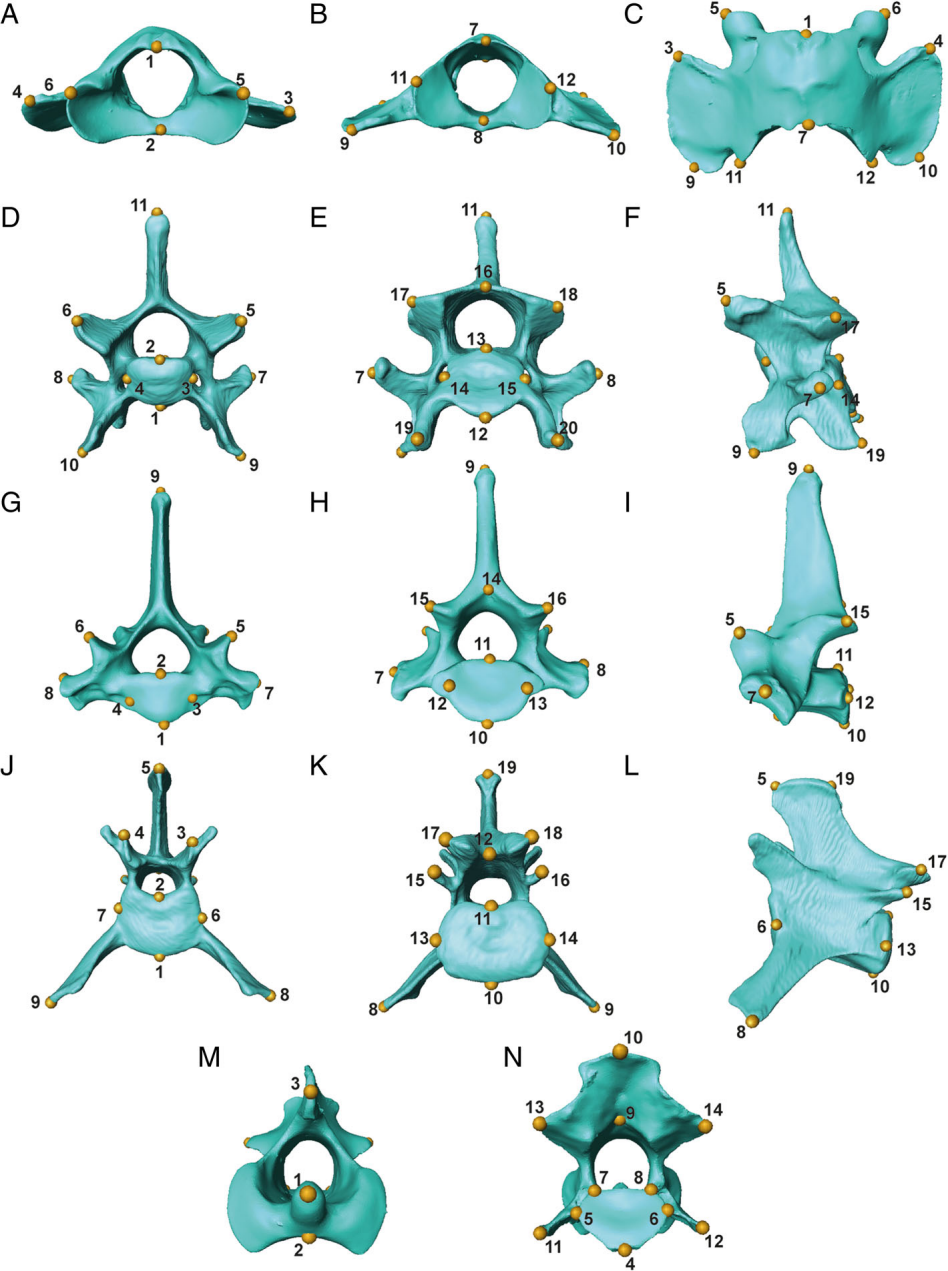
Examples of the different vertebral morphologies across the presacral vertebral column of felids and their respective three-dimensional landmarks. Each of the vertebrae shown here is a representative of a unique shape or possesses the maximum number of landmarks per morphology (i.e. the unique C1 and C2, an example of the cervical morphology with C6, T1 demonstrating the thoracic morphology, and L1 showing the lumbar morphology): **a**-**c** atlas (C1) in anterior, posterior and dorsal view; **d**-**f** C6 in anterior, posterior and lateral view; **g**-**i** T1 in anterior, posterior and lateral view; **j**-**l** L1 in anterior, posterior and lateral view; and **m**-**n** axis (C2) in anterior and posterior view. Vertebral images are from CT scans of *Acinonyx jubatus* (Cheetah, USNM 520539). Vertebra-specific landmark descriptions can be found in Additional file 1: Table S1

Following the methods outlined in our previous study [40], different sets of landmarks were collected per specific vertebrae due to differences in vertebral morphology throughout the axial skeleton: 12 landmarks were gathered on C1 (atlas), 14 on C2 (axis), 18 on C4, 20 on C6, 16 on C7 – T10, 16 on T11, 17 on T12 – T13, 19 on L1 – L4, and 17 on L6 – L7 (Fig. 3, and see Additional file 1: Tables S1 and S2 for landmarks identity). The chosen landmarks have been analysed in previous publications [31, 40], and have been shown to accurately describe the main aspects of vertebral shape, both when whole vertebral morphology was considered and regarding smaller landmark-module sets within individual vertebra. Furthermore, shape analyses of this data showed that it was able to capture morphological changes correlated with ecological specialisation in felids [40]. These landmarks were collected on 66 complete specimens of nine felid species (*Acinonyx jubatus, Felis catus, Leopardus pardalis, Leptailurus serval, Neofelis nebulosa, Panthera leo, Panthera pardus, Prionailurus bengalensis* and *Puma concolor*; Additional file 1: Table S3 for specimen numbers). The final dataset was therefore composed of 1254 individual vertebrae. The subset of nine species studied here include representatives of the ecological specialisations that have been described for Felidae (i.e., locomotion and prey size specialisations; [33, 36–39, 52]). Within this family, species vary in locomotor specialisation, including cursorial (e.g. *Acinonyx jubatus*), terrestrial (e.g. *Panthera leo*), scansorial (e.g. *Panthera pardus*) and arboreal (e.g. *Neofelis nebulosa*) species. With regards to specialisation in prey size, felids range from small prey specialists (<15 kg; e.g. *Felis catus*) to large prey specialists (>25Kg; e.g. *Puma concolor*), with a few species being less specialised and killing prey depending more on availability (mixed prey size; e.g. *Leopardus pardalis*). In addition to ecological specialisation, the species chosen for this study also represent the range in body mass observed in extant members of the family (e.g. from circa 3 kg in the domestic cat, *Felis catus*, to over 200 kg in the lion (*Panthera leo*) [36, 39].

### Data analysis

Analyses were carried out in R version 3.2.3 [53], using the ‘geomorph’ [54, 55] package. Prior to subsequent analyses, the landmark data for each vertebral type (i.e. vertebrae grouped by vertebral position, e.g. C1, C2, C4, T1, etc.) was separately aligned with a Generalised Procrustes Superimposition (GPA) in order to remove effects of scale, translation and rotation. The stability of the covariance matrices for each vertebrae was assessed by bootstrapping each dataset 10,000 times and comparing the covariance matrices of the original and resampled dataset with random skewers analysis [1, 56]. This analysis demonstrated that covariance matrix repeatability was high, ranging from 0.91 to 0.96 with a median of 0.94 and thus our sampling was sufficient for accurately estimating vertebral covariance matrices.

#### Vertebral shape covariation

The degree of morphological integration (i.e. shape covariation) per each possible pairwise combination between the vertebrae included here (e.g. C1 and C2, C1 and C4, C2 and C4 etc.) was measured using a two-block partial least square (PLS) analysis [57, 58]. This analysis was performed while accounting for phylogenetic relatedness, and was quantified by following these steps: first, landmark data for each vertebral type (e.g. T10) was separated into single species sets (e.g. *Panthera leo* only), which were individually aligned with a GPA. Species means per each vertebral type were then calculated from these Procrustes coordinates. Finally, pairwise mean vertebral shape covariation was estimated with a phylogenetic PLS, under a Brownian motion model of evolution [59]. Phylogenetic relationships between the species studied here were calculated using a pruned version of the composite tree by Piras et al. [60]. Statistical significance of each pairwise integration test was evaluated against a null distribution generated by repeating the phylogenetic PLS analysis after randomly permuting specimen rows for one vertebral dataset. Repeating this procedure with 5000 iterations generated the distribution against which the significance of the original results were compared.

There is some discussion of whether phylogeny should be corrected for when analysing patterns of integration, as removing this signal might conceal real genetic or developmental integration [61]. However, the phylogenetic PLS methodology used [59] has been widely accepted (e.g. [62–64]), and the application of this correction here reveals the major patterns of vertebral column organisation even in a highly conservative scenario.

The estimated degree of integration (i.e. covariation between pairs of vertebrae) and the statistical significance of each test (*p*-value; significance cut-off used a *p*-value <0.05 threshold, but see below) were then compiled in matrices where sets of vertebrae showing significant shape covariation (i.e. modules) could be visualised.

#### Covariation across centrum versus neural spine modules throughout the vertebral column

A second phylogenetic PLS analysis was carried out using the Procrustes-aligned mean species coordinates for landmarks present in the centrum or neural spine modules only (see Additional file 1: Table S2 for landmarks identity). Landmark assignment to these modules was based on developmental origins of vertebral components in amniotes [42] and ossification centres in felids [27]. Additionally, analysis of intravertebral morphological modularity across felids has shown that this model is supported in most presacral vertebrae [40].

#### Multiple comparisons and statistical significance

Because each individual vertebrae was involved in multiple comparisons, the significance test results (i.e. *p*-values) of each of the PLS analyses were corrected using a Benjamini-Hochberg procedure with a false discovery rate at 0.05, a relatively strict value [65]. The Benjamini-Hochberg correction is a method for taking into account false positives (i.e. cases in which the raw *p*-value is below the chosen threshold, e.g. 0.05, purely due to chance) in multiple comparisons analyses. We chose to use this procedure instead of the more common Bonferroni correction due to the latter method’s tendency to find a sizeable number of false negatives in analyses that include a large number of comparisons (e.g. a Bonferroni-corrected significance test for an analysis containing 171 comparisons, such as the one presented here, at an initial significance threshold of *p*-value <0.05, would entail that only *p*-values <0.0003 were to be considered significant) [65]. The way in which the Benjamini-Hochberg method classifies *p*-values according to their significance is by using a ranking system. First, all raw *p*-values are ordered from smallest to largest and ranked from *i* = 1 (lowest) to *m* = the total number of tests. These ranked raw *p*-values are then compared to their ‘Benjamini-Hochberg critical values, calculated as (*i/m*)*Q*, where Q is the chosen false discovery rate (0.05 here). The largest *p*-value which is still lower than their critical value plus all other lower raw *p*-values are classified as significant [65, 66]. This method also calculates Benjamini-Hochberg’-corrected *p*-values for easier visualisation, which are displayed here along with the raw *p*-values.

#### Allometry and vertebral integration

Allometry in vertebral shape was not corrected for prior to the analyses of intervertebral integration. Allometric shape changes (i.e. those directly driven by changes in body size) have been suggested to be a strong driver contributing towards morphological integration, particularly when analyses are performed between partitions within a single structure, because allometric effects may integrate a single structure uniformly [1, 9, 67]. However, our previous work on vertebral shape in the species studied here [31] has demonstrated that allometry varies across the presacral vertebral column, but only explain around 11% of vertebral shape differences across felids (mean 11.1%, median 9.9%). Further, body mass evolution in felids has been shown to be highly dependent on phylogenetic relationships [34, 36, 39], therefore correcting for size after having applied the phylogenetic correction performed here could potentially overcorrect and introduce error into our analyses. Finally, keeping in mind that the aim of this study was to investigate patterns of integration across the vertebral column, correcting for a factor that may be one of the constituents of such integration would potentially obscure real biological patterns of covariation between the vertebrae studied here.

## Additional file

Additional file 1. Table S1: Landmark number and description per vertebra. **Table S2:**Summary of landmarks composing each developmental module organisation of vertebral organisation, following [40]. **Table S3:** Specimen number information per species for the individuals used in the analyses presented here. Museum abbreviations are as follows: NHM: Natural History Museum, London; MNHN: Muséum National d’Histoire Naturelle, Paris; MCZ: Harvard Museum of Natural History, Cambridge; AMNH: American Museum of Natural History, New York; FMNH: Museum of Natural History, Chicago; USNM: Smithsonian National Museum of Natural History, Washington D.C. **Table S4:** Above diagonal cells display the *P* values for the pairwise covariation values from the phylogenetic PLS analysis of all landmarks’ coordinates. Below diagonal values show the *P* values after Benjamini-Hochberg correction. Results in bold and with grey shaded cells are significant (*P* < 0.05). **Table S5:** Above diagonal cells display the *P* values for the pairwise covariation values from the phylogenetic PLS analysis of centrum-only coordinates. Below diagonal values show the *P* values after Benjamini-Hochberg correction. Results in bold and with grey shaded cells are significant (*P* < 0.05). **Table S6:** Above diagonal cells display the *P* values for the pairwise covariation values from the phylogenetic PLS analysis of neural spine-only coordinates. Below diagonal values show the *P* values after Benjamini-Hochberg correction. Results in bold and with grey shaded cells are significant (*P* < 0.05). (DOCX 54 kb)
