# Supplementary material for: Morphological modularity in the vertebral column of Felidae (Mammalia, Carnivora)
Source: BMC Evol Biol. 2017 Jun 9;17:133. doi: 10.1186/s12862-017-0975-2 (PMC5466766; doi:10.1186/s12862-017-0975-2)
Supplement: Additional file 1. Table S1: — Landmark number and description per vertebra. Table S2:Summary of landmarks composing each developmental module organisation of vertebral organisation, following [40]. Table S3: Specimen number information per species for the individuals used in the analyses presented here. Museum abbreviations are as follows: NHM: Natural History Museum, London; MNHN: Muséum National d’Histoire Naturelle, Paris; MCZ: Harvard Museum of Natural History, Cambridge; AMNH: American Museum of Natural History, New York; FMNH: Museum of Natural History, Chicago; USNM: Smithsonian National Museum of Natural History, Washington D.C. Table S4: Above diagonal cells display the P values for the pairwise covariation values from the phylogenetic PLS analysis of all landmarks’ coordinates. Below diagonal values show the P values after Benjamini-Hochberg correction. Results in bold and with grey shaded cells are significant (P < 0.05). Table S5: Above diagonal cells display the P values for the pairwise covariation values from the phylogenetic PLS analysis of centrum-only coordinates. Below diagonal values show the P values after Benjamini-Hochberg correction. Results in bold and with grey shaded cells are significant (P < 0.05). Table S6: Above diagonal cells display the P values for the pairwise covariation values from the phylogenetic PLS analysis of neural spine-only coordinates. Below diagonal values show the P values after Benjamini-Hochberg correction. Results in bold and with grey shaded cells are significant (P < 0.05). (DOCX 54 kb) [file 12862_2017_975_MOESM1_ESM.docx]

Additional file 1

Table S1: Landmark number and description per vertebra.

| **Vertebra** | **Landmark** | **Description** |
| --- | --- | --- |
| Atlas | 1 | Anterior mid-point of dorsal arch |
|  | 2 | Anterior mid-point of ventral arch |
|  | 3 | Anterior lateral-most tip of left transverse process |
|  | 4 | Anterior lateral-most tip of right transverse process |
|  | 5 | Dorso-anterior-most tip of left pre-zygapophysis |
|  | 6 | Dorso-anterior-most tip of right pre-zygapophysis |
|  | 7 | Posterior mid-point of dorsal arch |
|  | 8 | Posterior mid-point of ventral arch |
|  | 9 | Posterior lateral-most tip of left transverse process |
|  | 10 | Posterior lateral-most tip of right transverse process |
|  | 11 | Posterior-most tip of left post-zygapophysis |
|  | 12 | Posterior-most tip of right post-zygapophysis |
| Axis | 1 | Anterior-most point at tip of den |
|  | 2 | Ventral mid-point at base of den |
|  | 3 | Anterior-most point of neural spine |
|  | 4 | Posterior ventral mid-point of centrum |
|  | 5 | Posterior left lateral-most point of width of centrum |
|  | 6 | Posterior right lateral-most point of width of centrum |
|  | 7 | Posterior left dorso-lateral point of centrum |
|  | 8 | Posterior right dorso-lateral point of centrum |
|  | 9 | Posterior dorsal mid-point of the neural canal |
|  | 10 | Dorsal posterior-most point at tip of neural spine |
|  | 11 | Left lateral-most posterior tip of transverse process |
|  | 12 | Right lateral-most posterior tip of transverse process |
|  | 13 | Posterior-most dorsal point of left post-zygapophysis |
|  | 14 | Posterior-most dorsal point of right post-zygapophysis |
| C4 | 1 | Anterior ventral mid-point of centrum |
|  | 2 | Anterior dorsal mid-point of centrum |
|  | 3 | Anterior left lateral-most point of centrum |
|  | 4 | Anterior left lateral-most point of centrum |
|  | 5 | Anterior dorsal-most point of left pre-zygapophyses |
|  | 6 | Anterior dorsal-most point of right pre-zygapophyses |
|  | 7 | Anterior-most point of left lamina |
|  | 8 | Anterior-most point of right lamina |
|  | 9 | Dorsal-most point at tip of neural spine |
|  | 10 | Posterior ventral mid-point of centrum |
|  | 11 | Posterior dorsal mid-point of centrum |
|  | 12 | Posterior Left lateral-most point of centrum |
|  | 13 | Posterior right lateral-most point of centrum |
|  | 14 | Posterior dorsal mid-point of the neural canal |
|  | 15 | Posterior-most point of left post-zygapophyses |
|  | 16 | Posterior-most point of right post-zygapophyses |
|  | 17 | Lateral-most point of left transverse process |
|  | 18 | Lateral-most point of right transverse process |
| C6 | 1 | Anterior ventral mid-point of centrum |
|  | 2 | Anterior dorsal mid-point of centrum |
|  | 3 | Anterior left lateral-most point of centrum |
|  | 4 | Anterior left lateral-most point of centrum |
|  | 5 | Anterior dorsal-most point of left pre-zygapophyses |
|  | 6 | Anterior dorsal-most point of right pre-zygapophyses |
|  | 7 | Lateral-most point of left transverse process |
|  | 8 | Lateral-most point of right transverse process |
|  | 9 | Anterior-most point of left lamina |
|  | 10 | Anterior-most point of right lamina |
|  | 11 | Dorsal-most point at tip of neural spine |
|  | 12 | Posterior ventral mid-point of centrum |
|  | 13 | Posterior dorsal mid-point of centrum |
|  | 14 | Posterior Left lateral-most point of centrum |
|  | 15 | Posterior right lateral-most point of centrum |
|  | 16 | Posterior dorsal mid-point of the neural canal |
|  | 17 | Posterior-most point of left post-zygapophyses |
|  | 18 | Posterior-most point of right post-zygapophyses |
|  | 19 | Posterior-most point of left lamina |
|  | 20 | Posterior-most point of right lamina |
| C7 - T10* | 1 | Anterior ventral mid-point of centrum |
|  | 2 | Anterior dorsal mid-point of centrum |
|  | 3 | Anterior left lateral-most point of centrum |
|  | 4 | Anterior left lateral-most point of centrum |
|  | 5 | Anterior dorsal-most point of left pre-zygapophysis |
|  | 6 | Anterior dorsal-most point of right pre-zygapophysis |
|  | 7 | Lateral-most point of left transverse process |
|  | 8 | Lateral-most point of right transverse process |
|  | 9 | Dorsal-most point at tip of neural spine |
|  | 10 | Posterior ventral mid-point of centrum |
|  | 11 | Posterior dorsal mid-point of centrum |
|  | 12 | Posterior Left lateral-most point of centrum |
|  | 13 | Posterior right lateral-most point of centrum |
|  | 14 | Posterior dorsal mid-point of the neural canal |
|  | 15 | Posterior-most point of left post-zygapophysis |
|  | 16 | Posterior-most point of right post-zygapophysis |
| T11 | 1 | Anterior ventral mid-point of centrum |
|  | 2 | Anterior dorsal mid-point of centrum |
|  | 3 | Anterior left lateral-most point of centrum |
|  | 4 | Anterior left lateral-most point of centrum |
|  | 5 | Anterior dorsal-most point of left pre-zygapophysis |
|  | 6 | Anterior dorsal-most point of right pre-zygapophysis |
|  | 7 | Posterior-most point of tip of left accessory process |
|  | 8 | Posterior-most point of tip of right accessory process |
|  | 9 | Dorsal-most point at tip of neural spine |
|  | 10 | Posterior ventral mid-point of centrum |
|  | 11 | Posterior dorsal mid-point of centrum |
|  | 12 | Posterior Left lateral-most point of centrum |
|  | 13 | Posterior right lateral-most point of centrum |
|  | 14 | Posterior dorsal mid-point of the neural canal |
|  | 15 | Posterior-most point of left post-zygapophysis |
|  | 16 | Posterior-most point of right post-zygapophysis |
| T12 - T13 | 1 | Anterior ventral mid-point of centrum |
|  | 2 | Anterior dorsal mid-point of centrum |
|  | 3 | Anterior left lateral-most point of centrum |
|  | 4 | Anterior left lateral-most point of centrum |
|  | 5 | Anterior dorsal-most point of left pre-zygapophyses |
|  | 6 | Anterior dorsal-most point of right pre-zygapophyses |
|  | 7 | Anterior Dorsal-most point at tip of neural spine |
|  | 8 | Posterior Dorsal-most point at tip of neural spine |
|  | 9 | Posterior ventral mid-point of centrum |
|  | 10 | Posterior dorsal mid-point of centrum |
|  | 11 | Posterior Left lateral-most point of centrum |
|  | 12 | Posterior right lateral-most point of centrum |
|  | 13 | Posterior dorsal mid-point of the neural canal |
|  | 14 | Posterior-most point of left post-zygapophyses |
|  | 15 | Posterior-most point of right post-zygapophyses |
|  | 16 | Posterior-most point of tip of left accessory process |
|  | 17 | Posterior-most point of tip of right accessory process |
| L1 - L4 | 1 | Anterior ventral mid-point of centrum |
|  | 2 | Anterior dorsal mid-point of centrum |
|  | 3 | Anterior dorsal-most point of left pre-zygapophyses |
|  | 4 | Anterior dorsal-most point of right pre-zygapophyses |
|  | 5 | Dorsal anterior-most point at tip of neural spine |
|  | 6 | Anterior left lateral-most point of centrum |
|  | 7 | Anterior left lateral-most point of centrum |
|  | 8 | Lateral-most point of left transverse process |
|  | 9 | Lateral-most point of right transverse process |
|  | 10 | Posterior ventral mid-point of centrum |
|  | 11 | Posterior dorsal mid-point of centrum |
|  | 12 | Posterior dorsal mid-point of the neural canal |
|  | 13 | Posterior Left lateral-most point of centrum |
|  | 14 | Posterior right lateral-most point of centrum |
|  | 15 | Posterior-most point of tip of left accessory process |
|  | 16 | Posterior-most point of tip of right accessory process |
|  | 17 | Posterior-most point of left post-zygapophyses |
|  | 18 | Posterior-most point of right post-zygapophyses |
|  | 19 | Dorsal posterior-most point at tip of neural spine |
| L6 - L7 | 1 | Anterior ventral mid-point of centrum |
|  | 2 | Anterior dorsal mid-point of centrum |
|  | 3 | Anterior dorsal-most point of left pre-zygapophyses |
|  | 4 | Anterior dorsal-most point of right pre-zygapophyses |
|  | 5 | Dorsal anterior-most point at tip of neural spine |
|  | 6 | Anterior left lateral-most point of centrum |
|  | 7 | Anterior left lateral-most point of centrum |
|  | 8 | Lateral-most point of left transverse process |
|  | 9 | Lateral-most point of right transverse process |
|  | 10 | Posterior ventral mid-point of centrum |
|  | 11 | Posterior dorsal mid-point of centrum |
|  | 12 | Posterior dorsal mid-point of the neural canal |
|  | 13 | Posterior Left lateral-most point of centrum |
|  | 14 | Posterior right lateral-most point of centrum |
|  | 15 | Posterior-most point of left post-zygapophyses |
|  | 16 | Posterior-most point of right post-zygapophyses |
|  | 17 | Dorsal posterior-most point at tip of neural spine |

Table S2: Summary of landmarks composing each developmental module organisation of vertebral organisation, following Chapter 4 (Randau and Goswami in press).

|  | CENTRUM MODULE | NEURAL-SPINE MODULE |
| --- | --- | --- |
| Atlas | 2; 8 | 1; 3 – 7; 9 - 12 |
| Axis  (3 modules) | 1, 2, 4 - 8 | 3; 9 – 14 |
| C4 | 1 – 4; 10 – 13 | 5 – 9; 14 – 18 |
| C6 | 1 – 4; 12 – 15 | 5 – 11; 16 – 20 |
| C7 – T10 | 1 – 4; 10 – 13 | 5 – 9; 14 – 16 |
| T11 | 1 – 4; 8 – 11 | 5 – 7; 12 – 16 |
| T12 – T13 | 1 – 4; 9 – 12 | 5 – 8; 13 – 17 |
| L1 – L4 | 1; 2; 6; 7; 10; 11; 13; 14 | 3 – 5; 8; 9; 12; 15 – 19 |
| L6 – L7 | 1; 2; 6; 7; 10; 11; 13; 14 | 3 – 5; 8; 9; 12; 15 – 17 |
|  |  |  |

Table S5.3: Specimen number information per species for the individuals used in the analyses presented here. Museum abbreviations are as follows: NHM: Natural History Museum, London; MNHN: Muséum National d’Histoire Naturelle, Paris; MCZ: Harvard Museum of Natural History, Cambridge; AMNH: American Museum of Natural History, New York; FMNH: Museum of Natural History, Chicago; USNM: Smithsonian National Museum of Natural History, Washington D.C.

| **Species** | **Specimen number** |
| --- | --- |
| Acinonyx jubatus | FMNH127834 |
|  | FMNH57826 |
|  | USNM520539 |
|  | AMNH119655 |
|  | AMNH119657 |
|  | AMNH119656 |
|  | AMNH36426 |
| Felis catus | USNM 396268 |
|  | USNM 396392 |
|  | USNM 397631 |
|  | USNM 398871 |
|  | USNM A21665 |
|  | NHM 1952 10 20 4 |
|  | NHM 1988 1 |
| Leopardus pardalis | FMNH 93174 |
|  | FMNH 68895 |
|  | USNM 271094 |
|  | USNM A14182 |
|  | MNHN 1998 1866 |
|  | MNHN A3456 |
|  | AMNH 214744 |
|  | AMNH 248728 |
| Leptailurus serval | FMNH 127843 |
|  | FMNH 44438 |
|  | FMNH 60491 |
|  | USNM 548666 |
|  | NHM 1855 6 30 2 |
|  | NHM 1845 9 25 23 |
|  | AMNH 34767 |
| Neofelis nebulosa | FMNH 54304 |
|  | USNM 399291 |
|  | USNM 545387 |
|  | MNHN 1961 217 |
|  | MNHN 1980 16 |
|  | NHM 1854 6 14 2 |
|  | NHM 1965 1 18 1 |
|  | AMNH 35273 |
| Panthera leo | FMNH 49340 |
|  | USNM 172677 |
|  | MCZ 9487 |
|  | AMNH 85147 |
| Panthera pardus | USNM 15684 |
|  | USNM 303320 |
|  | MNHN 1892 1079 |
|  | MNHN A13045 1844 |
|  | MNHN 1898 100 |
|  | MNHN 1906 454 |
|  | MNHN 1945 70 |
|  | MNHN A7932 |
|  | MNHN BII 4 |
|  | MNHN CG1998 582 |
|  | AMNH 54462 |
| Prionailurus bengalensis | FMNH 99363 |
|  | FMNH 121228 |
|  | USNM 317283 |
|  | NHM 1309b |
|  | NHM 77 2896 |
|  | NHM 1979 2895 |
|  | NHM 1309b 1858 |
| Puma concolor | FMNH129339 |
|  | USNM A21528 |
|  | USNM264166 |
|  | MNHN1937 4 |
|  | AMNH181997 |
|  | AMNH90213 |
|  | AMNH10259 |

Table S4: Above diagonal cells display the P values for the pairwise covariation values from the phylogenetic PLS analysis of all landmarks’ coordinates. Below diagonal values show the P values after Benjamini-Hochberg correction. Results in bold and with grey shaded cells are significant (P < 0.05).

|  | **ATLAS** | **AXIS** | **C4** | **C6** | **C7** | **T1** | **T2** | **T4** | **T6** | **T8** | **T10** | **T11** | **T12** | **T13** | **L1** | **L2** | **L4** | **L6** | **L7** |
| --- | --- | --- | --- | --- | --- | --- | --- | --- | --- | --- | --- | --- | --- | --- | --- | --- | --- | --- | --- |
| **ATLAS** | 1 | **0.001** | *0.051* | **0.014** | **0.03** | **0.033** | *0.132* | *0.119* | **0.023** | **0.004** | *0.155* | **0.036** | **0.014** | **0.039** | **0.03** | **0.024** | *0.08* | *0.052* | **0.023** |
| **AXIS** | **0.01** |  | **0.005** | **0.002** | **0.004** | **0.018** | *0.083* | *0.213* | *0.109* | **0.011** | **0.024** | **0.015** | **0.004** | **0.008** | **0.003** | **0.004** | **0.023** | **0.013** | **0.003** |
| **C4** | *0.108* | **0.022** | 1 | **0.001** | *0.087* | *0.192* | *0.141* | *0.249* | *0.497* | *0.316* | *0.623* | *0.722* | *0.256* | *0.133* | *0.075* | *0.085* | *0.128* | *0.147* | *0.114* |
| **C6** | **0.042** | **0.016** | **0.01** |  | **0.004** | **0.005** | **0.014** | *0.757* | *0.644* | *0.401* | *0.687* | *0.468* | **0.025** | **0.005** | **0.004** | **0.006** | **0.005** | **0.012** | *0.079* |
| **C7** | *0.071* | **0.02** | *0.163* | **0.02** | 1 | *0.050** | *0.13* | *0.528* | *0.267* | **0.04** | *0.322* | *0.41* | **0.001** | **0.001** | **0.002** | **0.001** | **0.004** | **0.002** | **0.001** |
| **T1** | *0.077* | *0.051* | *0.266* | **0.022** | *0.107* |  | **0.03** | *0.464* | *0.47* | *0.097* | **0.008** | **0.012** | *0.081* | *0.2* | *0.175* | *0.108* | *0.256* | *0.126* | **0.036** |
| **T2** | *0.212* | *0.161* | *0.219* | **0.042** | *0.212* | **0.03** | 1 | *0.663* | *0.444* | *0.132* | *0.249* | *0.227* | *0.626* | *0.642* | *0.433* | *0.514* | *0.529* | *0.496* | *0.285* |
| **T4** | *0.201* | *0.289* | *0.315* | *0.757* | *0.559* | **0.041** | *0.675* |  | **0.013** | **0.008** | *0.17* | **0.02** | *0.136* | *0.247* | *0.223* | *0.148* | *0.193* | *0.118* | *0.388* |
| **T6** | *0.06* | *0.19* | *0.537* | *0.66* | *0.331* | *0.161* | *0.502* | **0.041** | 1 | **0.018** | **0.012** | **0.008** | **0.043** | *0.094* | *0.169* | *0.144* | *0.232* | *0.191* | *0.479* |
| **T8** | **0.02** | **0.041** | *0.649* | *0.466* | *0.088* | *0.273* | *0.212* | **0.03** | *0.051* |  | *0.23* | *0.33* | **0.013** | *0.099* | *0.171* | *0.082* | *0.141* | *0.102* | *0.396* |
| **T10** | *0.228* | *0.061* | *0.727* | *0.695* | *0.39* | *0.071* | *0.315* | *0.243* | **0.041** | *0.302* | 1 | **0.001** | *0.163* | **0.034** | *0.458* | *0.533* | *0.384* | *0.142* | *0.107* |
| **T11** | *0.081* | **0.044** | *0.319* | *0.518* | *0.473* | *0.518* | *0.301* | *0.055* | **0.03** | *0.397* | **0.01** |  | *0.084* | *0.153* | *0.236* | *0.391* | *0.518* | *0.436* | *0.219* |
| **T12** | **0.042** | **0.02** | *0.212* | *0.063* | **0.01** | *0.518* | *0.649* | *0.215* | *0.093* | **0.041** | *0.238* | *0.161* | 1 | **0.003** | **0.019** | **0.012** | **0.013** | **0.007** | **0.027** |
| **T13** | *0.087* | **0.03** | *0.386* | **0.022** | **0.01** | *0.178* | *0.66* | *0.315* | *0.175* | *0.18* | *0.078* | *0.227* | **0.01** |  | **0.001** | **0.001** | **0.002** | **0.003** | **0.006** |
| **L1** | *0.071* | **0.019** | *0.154* | **0.02** | **0.016** | *0.247* | *0.496* | *0.298* | *0.243* | *0.243* | *0.515* | *0.305* | **0.01** | **0.001** | 1 | **0.001** | **0.002** | **0.003** | **0.001** |
| **L2** | *0.061* | **0.02** | *0.161* | **0.026** | **0.01** | *0.19* | *0.553* | *0.222* | *0.22* | *0.161* | *0.56* | *0.461* | **0.016** | **0.001** | 0.01 | 1 | **0.001** | **0.001** | **0.001** |
| **L4** | *0.161* | *0.06* | *0.212* | **0.022** | **0.02** | *0.319* | *0.559* | *0.266* | *0.303* | *0.219* | *0.459* | *0.554* | **0.019** | **0.002** | 0.016 | 0.01 | 1 | **0.001** | **0.001** |
| **L6** | *0.108* | **0.041** | *0.222* | **0.041** | **0.016** | *0.211* | *0.537* | *0.201* | *0.266* | *0.183* | *0.219* | *0.497* | **0.026** | **0.003** | 0.019 | 0.01 | **0.01** | 1 | **0.001** |
| **L7** | *0.06* | **0.019** | *0.197* | 0.161 | **0.01** | *0.081* | *0.35* | *0.46* | *0.525* | *0.463* | *0.19* | *0.295* | 0.067 | **0.006** | 0.01 | 0.01 | **0.01** | **0.01** | 1 |

Table S5: Above diagonal cells display the P values for the pairwise covariation values from the phylogenetic PLS analysis of centrum-only coordinates. Below diagonal values show the P values after Benjamini-Hochberg correction. Results in bold and with grey shaded cells are significant (P < 0.05).

|  | **ATLAS** | **AXIS** | **C4** | **C6** | **C7** | **T1** | **T2** | **T4** | **T6** | **T8** | **T10** | **T11** | **T12** | **T13** | **L1** | **L2** | **L4** | **L6** | **L7** |
| --- | --- | --- | --- | --- | --- | --- | --- | --- | --- | --- | --- | --- | --- | --- | --- | --- | --- | --- | --- |
| **ATLAS** | 1 | **0.005** | **0.018** | **0.02** | **0.011** | **0.008** | **0.036** | *0.054* | *0.088* | **0.025** | **0.02** | **0.016** | **0.005** | **0.047** | **0.031** | **0.032** | *0.08* | *0.079* | *0.085* |
| **AXIS** | **0.019** | 1 | **0.008** | **0.019** | **0.001** | **0** | **0.012** | *0.055* | *0.072* | **0.019** | **0.03** | *0.067* | **0.029** | **0.048** | **0.005** | **0.01** | **0.019** | *0.07* | **0.05*** |
| **C4** | **0.042** | **0.025** | 1 | **0.001** | **0.005** | *0.086* | **0.017** | *0.126* | *0.115* | **0.015** | **0.022** | *0.097* | **0.045** | **0.037** | **0.018** | **0.033** | **0.038** | *0.088* | *0.218* |
| **C6** | **0.042** | **0.042** | **0.006** | 1 | *0.161* | *0.468* | **0.032** | *0.322* | *0.327* | *0.125* | **0.024** | *0.188* | *0.24* | *0.214* | **0.018** | *0.133* | *0.073* | *0.089* | *0.121* |
| **C7** | **0.031** | **0.006** | **0.019** | *0.179* | 1 | **0.002** | **0.019** | **0.042** | **0.043** | **0.004** | **0.007** | **0.01** | **0.001** | **0.001** | **0** | **0** | **0** | **0** | **0.007** |
| **T1** | **0.025** | **0** | *0.109* | *0.468* | **0.01** | 1 | *0.059* | *0.148* | *0.072* | **0.027** | *0.199* | *0.095* | *0.121* | *0.128* | **0.025** | **0.038** | **0.05*** | *0.084* | *0.126* |
| **T2** | *0.062* | **0.033** | **0.042** | *0.056* | **0.042** | *0.083* | 1 | *0.345* | *0.378* | **0.049** | *0.345* | *0.164* | *0.09* | *0.13* | *0.342* | *0.208* | *0.262* | *0.334* | *0.059* |
| **T4** | *0.079* | *0.079* | *0.146* | *0.336* | *0.07* | *0.167* | *0.349* | 1 | **0.015** | **0.001** | *0.055* | **0.048** | *0.054* | *0.069* | *0.089* | *0.065* | *0.054* | **0.012** | *0.197* |
| **T6** | *0.109* | *0.095* | *0.138* | *0.339* | *0.071* | *0.095* | *0.38* | **0.038** | 1 | **0** | **0.031** | **0.003** | **0.023** | **0.016** | **0.026** | **0.021** | **0.046** | **0.014** | *0.065* |
| **T8** | **0.048** | **0.042** | **0.038** | *0.146* | **0.018** | *0.051* | *0.075* | **0.006** | **0** | 1 | **0.048** | **0.011** | **0.046** | **0.02** | **0.025** | **0.013** | **0.019** | **0.005** | *0.155* |
| **T10** | **0.042** | *0.055* | **0.045** | **0.048** | **0.023** | *0.215* | *0.349* | *0.079* | *0.056* | *0.074* | 1 | **0.011** | **0.002** | **0.003** | **0.001** | **0.002** | **0.001** | **0.006** | **0.014** |
| **T11** | **0.04** | *0.092* | *0.117* | *0.206* | **0.03** | *0.115* | *0.181* | *0.074* | **0.014** | **0.031** | **0.031** | 1 | **0.002** | **0.001** | **0.004** | **0.006** | **0.01** | **0.008** | *0.07* |
| **T12** | **0.019** | *0.054* | *0.073* | *0.253* | **0.006** | *0.143* | *0.11* | *0.079* | **0.046** | *0.074* | **0.01** | **0.01** | 1 | **0** | **0** | **0** | **0** | **0.001** | **0.002** |
| **T13** | *0.074* | *0.074* | *0.063* | *0.229* | **0.006** | *0.147* | *0.148* | *0.094* | **0.04** | **0.042** | **0.014** | **0.006** | **0** | 1 | **0** | **0** | **0** | **0** | **0.004** |
| **L1** | *0.056* | **0.019** | **0.042** | **0.042** | **0** | **0.048** | *0.349* | *0.109* | **0.049** | **0.048** | **0.006** | **0.018** | **0** | **0** | 1 | **0** | **0.001** | **0** | **0.006** |
| **L2** | *0.056* | **0.03** | *0.058* | *0.151* | **0** | *0.064* | *0.224* | *0.09* | **0.044** | **0.035** | **0.01** | **0.021** | **0** | **0** | **0** | 1 | **0** | **0** | **0.005** |
| **L4** | *0.104* | **0.042** | *0.064* | *0.096* | **0** | *0.075* | *0.275* | *0.079* | *0.074* | **0.042** | **0.006** | **0.03** | **0** | **0** | **0.006** | **0** | 1 | **0** | **0.005** |
| **L6** | *0.103* | *0.094* | *0.109* | *0.109* | **0** | *0.108* | *0.344* | **0.033** | **0.037** | **0.019** | **0.021** | **0.025** | **0.006** | **0** | **0** | **0** | **0** | 1 | **0.022** |
| **L7** | *0.108* | *0.075* | *0.232* | *0.143* | **0.023** | *0.146* | *0.083* | *0.215* | *0.09* | *0.173* | **0.037** | *0.094* | **0.01** | **0.018** | **0.021** | **0.019** | **0.019** | **0.045** | 1 |

Table S6: Above diagonal cells display the P values for the pairwise covariation values from the phylogenetic PLS analysis of neural spine-only coordinates. Below diagonal values show the P values after Benjamini-Hochberg correction. Results in bold and with grey shaded cells are significant (P < 0.05).

|  | **ATLAS** | **AXIS** | **C4** | **C6** | **C7** | **T1** | **T2** | **T4** | **T6** | **T8** | **T10** | **T11** | **T12** | **T13** | **L1** | **L2** | **L4** | **L6** | **L7** |
| --- | --- | --- | --- | --- | --- | --- | --- | --- | --- | --- | --- | --- | --- | --- | --- | --- | --- | --- | --- |
| **ATLAS** | 1 | **0** | *0.122* | **0.003** | **0.005** | **0.016** | **0.046** | *0.2* | *0.11* | *0.312* | **0.031** | *0.051* | **0.012** | **0.014** | **0.005** | **0.005** | **0.019** | **0.021** | **0.003** |
| **AXIS** | **0** | 1 | **0.022** | **0** | **0.005** | *0.168* | *0.199* | *0.311* | *0.129* | *0.2* | **0.012** | **0.012** | **0** | **0.012** | **0.009** | **0.002** | **0.021** | **0.005** | **0.006** |
| **C4** | *0.234* | *0.057* | 1 | **0.003** | *0.192* | *0.325* | *0.332* | *0.319* | *0.453* | *0.733* | *0.63* | *0.852* | *0.467* | *0.2* | *0.102* | *0.133* | *0.204* | *0.301* | *0.15* |
| **C6** | **0.017** | **0** | **0.017** | 1 | **0.004** | **0.005** | **0.004** | *0.788* | *0.606* | *0.739* | *0.722* | *0.485* | **0.004** | **0.002** | **0.002** | **0.002** | **0.004** | **0.012** | **0.037** |
| **C7** | **0.019** | **0.019** | *0.332* | **0.018** | 1 | *0.057* | *0.279* | *0.788* | *0.376* | *0.68* | *0.122* | *0.555* | **0.003** | **0.001** | **0.003** | **0** | **0.003** | **0.001** | **0.004** |
| **T1** | **0.045** | *0.296* | *0.434* | **0.019** | *0.123* | 1 | **0.01** | *0.588* | *0.571* | *0.159* | **0.026** | **0.035** | **0.038** | *0.164* | *0.193* | *0.107* | *0.256* | *0.09* | **0.031** |
| **T2** | *0.105* | *0.332* | *0.437* | **0.018** | *0.401* | **0.034** | 1 | *0.723* | *0.279* | *0.3* | *0.246* | *0.278* | *0.631* | *0.628* | *0.439* | *0.585* | *0.552* | *0.533* | *0.329* |
| **T4** | *0.332* | *0.427* | *0.43* | *0.802* | *0.802* | *0.675* | *0.763* | 1 | **0.012** | **0.044** | *0.122* | **0.045** | *0.249* | *0.425* | *0.351* | *0.249* | *0.296* | *0.219* | *0.606* |
| **T6** | *0.224* | *0.243* | *0.561* | *0.682* | *0.483* | *0.664* | *0.401* | **0.035** | 1 | *0.114* | **0.019** | **0.004** | *0.076* | *0.266* | *0.318* | *0.249* | *0.278* | *0.284* | *0.65* |
| **T8** | *0.427* | *0.332* | *0.764* | *0.766* | *0.727* | *0.286* | *0.418* | *0.103* | *0.229* | 1 | *0.131* | *0.26* | *0.263* | *0.525* | *0.476* | *0.387* | *0.502* | *0.422* | *0.256* |
| **T10** | *0.077* | **0.035** | *0.692* | *0.763* | *0.234* | *0.066* | *0.391* | *0.234* | *0.052* | *0.243* | 1 | **0.001** | *0.012* | *0.346* | *0.655* | *0.814* | *0.604* | *0.847* | *0.731* |
| **T11** | *0.113* | **0.035** | *0.852* | *0.588* | *0.65* | *0.086* | *0.401* | *0.104* | **0.018** | *0.397* | **0.01** | 1 | *0.116* | **0.049** | *0.773* | *0.13* | *0.674* | *0.625* | *0.277* |
| **T12** | **0.035** | **0** | *0.575* | **0.018** | **0.017** | *0.09* | *0.692* | *0.391* | *0.162* | *0.398* | **0.035** | *0.231* | 1 | **0.001** | **0.016** | **0.007** | **0.012** | **0.007** | *0.055* |
| **T13** | **0.041** | **0.035** | *0.332* | **0.014** | **0.01** | *0.292* | *0.692* | *0.534* | *0.399* | *0.628* | *0.452* | *0.11* | **0.01** | 1 | **0** | **0** | **0.003** | **0.001** | **0.006** |
| **L1** | **0.019** | **0.031** | *0.213* | **0.014** | **0.017** | *0.332* | *0.548* | *0.455* | *0.43* | *0.581* | *0.709* | *0.796* | **0.045** | **0** | 1 | **0** | **0.002** | **0.004** | **0.002** |
| **L2** | **0.019** | **0.014** | *0.245* | **0.014** | **0** | *0.22* | *0.675* | *0.391* | *0.391* | *0.494* | *0.824* | *0.243* | **0.025** | **0** | **0** | 1 | **0** | **0** | **0.001** |
| **L4** | *0.052* | *0.055* | *0.335* | **0.018** | **0.018** | *0.394* | *0.65* | *0.418* | *0.401* | *0.605* | *0.682* | *0.725* | **0.035** | **0.017** | **0.014** | **0** | 1 | **0** | **0.001** |
| **L6** | *0.055* | **0.019** | *0.418* | **0.035** | **0.01** | *0.19* | *0.633* | *0.357* | *0.405* | *0.534* | *0.852* | *0.692* | **0.025** | **0.01** | **0.018** | **0** | **0** | 1 | **0.001** |
| **L7** | **0.017** | **0.022** | *0.273* | *0.089* | **0.018** | *0.077* | *0.436* | *0.682* | *0.708* | *0.394* | *0.764* | *0.401* | *0.121* | **0.022** | **0.014** | **0.01** | **0.01** | **0.01** | 1 |
